# Novel Genes Associated with Dairy Traits in Sarda Sheep

**DOI:** 10.3390/ani11082207

**Published:** 2021-07-26

**Authors:** Michele Pazzola, Giuseppe Massimo Vacca, Pietro Paschino, Giovanni Bittante, Maria Luisa Dettori

**Affiliations:** 1 Department of Veterinary Medicine, University of Sassari, Via Vienna 2, 07100 Sassari, SS, Italy; pazzola@uniss.it (M.P.); ppaschino@uniss.it (P.P.); mldettori@uniss.it (M.L.D.); 2 Department of Agronomy, Food, Natural Resources, Animals and Environment (DAFNAE), University of Padova, Viale dell’Università 16, 35020 Legnaro, PD, Italy; bittante@unipd.it

**Keywords:** candidate gene, single nucleotide polymorphism, sheep milk, milk coagulation

## Abstract

**Simple Summary:**

In a population of 1112 individuals of Sarda sheep, we investigated the polymorphism of 45 single nucleotide polymorphisms (SNPs) in 15 different genes that have been previously investigated because of their relation to metabolism and innate immunity. For SNPs with a sufficient allele frequency, we also tested their influence on a wide panel of dairy traits. The genotyping evidenced some new genetic patterns, and seven SNPs had significant influences on the milk yield, composition and coagulation traits. The results may represent a starting point to acquire new knowledge in the field of dairy sheep sector and to improve the breeding schemes for the Sarda sheep breed.

**Abstract:**

The aim of the present research was to analyze the variability of 45 SNPs from different genes involved in metabolism and innate immunity to perform an association analysis with the milk yield, composition and milk coagulation traits. A population of 1112 Sarda breed sheep was sampled. Genotyping was generated by a TaqMan Open Array^TM^. Thirty out of the 45 SNPs were polymorphic, and 12 displayed a minor allele frequency higher than 0.05. An association analysis showed that the variability at genes *PRKAG3* and *CD14* was significantly associated with the daily milk yield. The variability at *PRKAG3* was also associated with the protein and casein content, somatic cell score and bacterial score. The variation at the *PRKAA2* gene was associated with the milk lactose concentration. The SNPs at *CD14* were also associated with the traditional milk coagulation properties, while the SNPs at GHR and GHRHR were associated with k_SR_, a derived coagulation parameter related to the rate of syneresis. The information provided here is new and increases our knowledge of genotype–phenotype interactions in sheep. Our findings might be useful in appropriate breeding schemes to be set up for the Sarda sheep breed, but these should be confirmed by further studies, possibly performed on independent populations.

## 1. Introduction

Sheep are farmed all over the world, because of their great adaptability and relatively high productions. Productive specialization is different among countries and geographical areas. In the Middle East and in the Mediterranean basin, sheep milk is of major importance [1]. In the European Union, sheep milk production has been markedly increased in the last decades, extending from 2 to about 3 million t in the period of 1960–2019, and dairy sheep farming is a relevant sector of agriculture in many areas, such as for Greece, the leading EU country with 944 thousand t of ewe milk produced in 2019, followed by Romania, Spain and Italy [2].

In dairy sheep farming, almost all milk is transformed into cheese; therefore, milk traits related to cheese-making, including milk coagulation properties (MCP), curd firming and syneresis, need to be evaluated, as they might impact the farm income [3]. The MCP are described as the rennet coagulation time (RCT), curd firming time (k_20_) and curd firmness after 30 min of analysis (a_30_), obtained by the lactodynamographic instrument [4]. The same analysis can be extended to 60 min with the additional single point measures of curd firmness at 45 (a_45_) and 60 (a_60_) minutes [5]. The data can be further analyzed by utilizing all the lactodynamographic records (one curd-firming data every 15 s) to set up the modeling equations of the coagulation process [6]. The derived parameters estimated by the modeling process are useful for understanding the entire milk coagulation process: the asymptotic potential curd firmness, CF_P_, the curd-firming instant rate constant, k_CF_, the syneresis instant rate constant, k_SR_, the maximum curd firmness value, CF_max_, and the time to attain the maximum curd firmness, t_max_ [6]. Traditional and derived MCP have been studied in cattle and, later, in sheep to provide important information on the dynamics of coagulation, highlighting the similarities and differences between species [6,7].

The molecular information underlying the biological processes has often been useful for understanding important phenotypes in sheep, such as prolificacy [8] or muscular hypertrophy [9]. The path to understand the molecular basis of the exceptional meatiness of the Texel sheep went through the identification of a quantitative trait locus on sheep chromosome 2 after a whole-genome scan and, finally, to the identification of a SNP in the 3′UTR of the *GDF8* (Growth Differentiation Factor 8) gene, which causes translational inhibition of the myostatin gene in sheep, thus contributing to muscular hypertrophy [9].

An association analysis, by correlating markers with phenotypes across a population, in addition to genome-wide association studies (based on whole-genome genotyping), and family-based linkage studies, might be useful to understand the role played by candidate genes or haplotypes in a population [10,11]. In the literature, many association studies investigating the ovine species (*Ovis aries*) are focused on growth, body weight and fat traits, as well as fertility and wool traits [12]. The research articles investigating sheep dairy traits are lower in number, and those mainly analyze casein and whey protein genes, as casein genes encode 80% of milk proteins (caseins), while whey proteins account for 20% of total milk proteins [13,14]. Noce et al. [13] investigated the polymorphism at the promoter regions of the four casein genes, in addition to the *LALBA* (alpha lactalbumin) and *BLG* (beta lactoglobulin) genes, and they highlighted significant associations with milk composition and coagulation properties in Sarda sheep [13]. Usai et al. [14] performed OvineSNP50 genotyping to map the genomic regions affecting milk traits and obtained a strong signal for the protein content on OAR chromosome 6, overlapping the region including the casein gene cluster in addition to OAR 4 for the fat content and OAR 11 for the milk yield traits.

Additionally, lactation is a complex phenomenon involving metabolic changes determined by many other genes, which ultimately, might affect the milk traits. The study by Bittante et al. [15] evidenced that, in sheep species, milk coagulation traits have moderate-to-high values of heritability, which can be exploitable in breeding programs. Hence, we hypothesize a role of some genes on the milk coagulation traits, based on their biological role of avoiding those genes that have already been studied and whose association with dairy traits has been demonstrated [12,13,14].

As regards the genes involved in cellular metabolism, *PRKAA2* (Protein Kinase AMP-Activated Catalytic Subunit Alpha2), *PRKAG3* (Protein Kinase AMP-Activated Non-Catalytic Subunit Gamma3) and the genes linked to the growth hormone system, such as *GHRHR* (Growth Hormone Releasing Hormone Receptor), *GHR* (Growth Hormone receptor) and *IGF1* (Insulin Like Growth Factor 1), the *PRKAA2* gene encodes a protein involved in the monitoring of the cellular energy status and, also, in response to stress [16]. The expression of *PRKAA2* changes throughout the different stages of pregnancy and lactation in cattle, suggesting a crucial role in the complex processes of lipolysis and milk synthesis [17,18]. *PRKAG3* is mainly studied for its role in regulating energy homeostasis and, also, in response to environmental or nutritional stress, mainly in skeletal muscle cells, where *PRKAG3* increases the oxidation of fatty acids and glucose uptake to meet the muscle energy demands [19]. The *PRKAG3* protein has been shown to affect the glycogen content in muscle cells and then the meat quality in livestock [20,21,22]. The growth hormone system plays an important role in protein and lipid metabolism and affects the galactopoiesis in dairy species [23]. We investigated the polymorphism at the *B4GALT1* (β1,4-galactosyltransferase 1) and *ABCG2* (ATP-binding cassette subfamily G member 2) genes. The β1,4-galactosyltransferase 1 protein is involved in glucose metabolism, as it forms a heterodimer with alpha lactalbumin in mammary epithelial cells. That generates the lactose synthase enzyme, which synthesizes lactose by combining one molecule of UDP-galactose with one of glucose [24]. The *ABCG2* protein belongs to a transporter family, including the proteins involved in the transport of molecules across cell membranes [25]. The *ABCG2* gene is expressed in different tissues, including the mammary gland epithelial cell during lactation, and plays an important role in active secretion of molecules across the blood–milk barrier, including drugs and toxins [26].

As regards the genes involved in the immune response and defense against pathogens, *CD14* (Cluster of Differentiation 14 Molecule), *TLR2* (Toll-Like Receptor 2), *TLR4* (Toll-Like Receptor 4), and *PI* (Serpin Peptidase Inhibitor), whose variability in dairy sheep and correlation with milk traits has not been investigated yet, the *CD14* gene encodes a molecule that is fundamental for innate immunity and acts against a range of pathogens. The *CD14* protein is a surface antigen that is preferentially expressed on monocytes and macrophages, which contributes to mediate the response to bacterial lipopolysaccharide [16]. The *TLR4* gene encodes a protein, a member of the Toll-like receptor family, involved in pathogen recognition and innate immunity. As the *TLR4* receptor is involved in the cellular response to the lipopolysaccharides found in most Gram-negative bacteria, it also plays a role in mastitis [27]. We also evaluated the variability at the Neuropeptide FF receptor 2 (*NPFFR2*) gene. NPFFR2 is a member of the G protein-coupled receptor superfamily, membrane proteins activated by neuropeptides NPAF and NPFF [28]. The NPFF neuropeptides were shown to display anti-inflammatory activity in a mouse model and were linked to T-lymphocyte proliferation and the immune response [29].

In addition to the above-mentioned metabolism and immune response-related genes, we investigated the variability of the *PLCE1* and *TYRP1* genes. The *PLCE1* (Phospholipase C Epsilon 1) gene encodes a phospholipase enzyme, which catalyzes the hydrolysis of phosphatidylinositol-4,5-bisphosphate, and produces the second messengers inositol 1,4,5-triphosphate (IP3) and diacylglycerol (DAG). The two second messengers can affect cell growth, differentiation, and gene expression [16]. The *TYRP1* gene (Tyrosinase Related Protein 1) plays an important role in the synthesis of eumelanin and is associated with the coat color in domestic animals, including sheep [30].

The present study aimed to explore, in a population of 1112 dairy ewes of the Sarda breed, the variability of 45 SNPs located on fifteen genes responsible for metabolism and immune defense reactions in order to perform an association analysis between the genotyped SNPs and a wide range of dairy traits, including milk yield and composition, as well as traditional and model coagulation traits.

## 2. Materials and Methods

### 2.1. Farms, Animals, and Samples

For the present study, 1112 ewes in 23 different farms were sampled. Detailed features of the animals and farms were reported by Pazzola et al. [5]. The main aspects were the following: ewes were of Sarda breed, regularly recorded in the flock book, and daughters of 120 different sires (minimum 4, maximum 40 daughters per sire); parity order and stage of lactation of ewes were, respectively, between the first and ninth, and from the second to the seventh month after parturition; farms were of commercial type, located in the regional territory of Sardinia (Italy) and managed under semi-extensive farming systems.

At each farm, a variable group of ewes were sampled (minimum 22, maximum 89 ewes per farm) in a single sampling day. Individual milk samples were collected in 200-mL sterile plastic containers during the evening milking, which was performed by using manually operated milking machines. The daily milk yield (dMY) was calculated as the sum of the morning and evening milking of the same sampling day. Blood samples were individually collected in K_3_EDTA vacuum tubes (BD Vacutainer, Becton Dickinson, Milan, Italy) from the jugular vein. Milk and blood samples were transported at 4 °C to the laboratories to be stored and analyzed.

### 2.2. DNA Extracton and Genotyping

Blood samples were processed for DNA extraction within 24 h after sampling. Genomic DNA was extracted using the Gentra Puregene blood kit (Qiagen, Hilden, Germany). DNA concentration and purity were assessed using an Eppendorf BioPhotometer (Eppendorf, Hamburg, Germany).

We choose each SNP to be analyzed in the present experiment, respecting the technical requirements of the TaqMan^®^ assay, and by using the Ensembl Genome Browser (https://www.ensembl.org/index.html; accessed date 30 March 2021) to localize and choose each SNP. The list of selected SNPs is reported in Supplementary Table S1. The selected SNPs and their flanking sequences of 60 bp (Supplementary Table S1) were submitted to the Custom TaqMan Assay Design Tool website (https://www5.appliedbiosystems.com/tools/cadt; accessed date 30 March 2021; Life Technologies Thermo Fisher Scientific, Waltham, MA, USA) to ascertain their suitability to be genotyped with the TaqMan Open Array multiplex assay.

The 12 K Flex QuantStudio instrument (Thermo Fisher Scientific, Waltham, MA, USA) was used for genotyping and Taqman Genotyper v.1.3 software (Applied Biosystems, Waltham, MA, USA) for visualizing the genotypes. Automatic allele calling was carried out using GeneCall software (Illumina, San Diego, CA, USA) with a CG threshold of 0.25 and a further analysis of the individual genotypes using SVS software version 7 (Golden Helix Inc., Bozeman, MT, USA). Samples or SNPs with call rates < 0.9 were removed from the dataset. The minor allele frequencies (MAF) observed and expected heterozygosity, consistency of the genotype distributions with the Hardy-Weinberg equilibrium, definition of the linkage disequilibrium (LD), and LD blocks were estimated by the Haploview software package [31].

### 2.3. Milk Analysis

Analysis of the milk samples was performed within 24 h after collection, as reported by Dettori et al. [32]. As regards the fat, protein, casein, lactose content, and pH, these were achieved by using the standard of the International Organization for Standardization and International Dairy Federation standard [33] and the MilkoScan FT6000 instrument (Foss Electric A/S, Hillerød, Denmark); the total bacterial count (TBC) was measured according to the ISO-IDF standard [34] by using a BactoScan FC150 instrument (Foss Electric); the somatic cell count (SCC) was measured according to the ISO-IDF method [35] by using a Fossomatic 5000 instrument (Foss Electric). The TBC and SCC were transformed into respective logarithmic scores to normalize the distribution: LBC (log-bacterial count = log_10_ (total bacterial count/1000), according to the ISO-IDF standard [36])) and SCS (somatic cell score = log_2_ (SCC × 10^−5^) + 3, according to Shook [37]).

As regards the coagulation traits, both the traditional milk coagulation properties (MCP) and curd firmness over time traits (CF_t_) were measured. The following MCPs were measured according to the method by McMahon and Brown [4] and the revisions for sheep species by Pazzola et al. [5] by using a Formagraph instrument (Foss Italia, Padova, Italy): RCT (rennet coagulation time in min); k_20_ (curd firming time in min); and a_30_, a_45_, and a_60_ (curd firmness 30, 45, and 60 min after rennet addition in mm). The following CF_t_ traits were measured according to a method used for ovine species by Vacca et al. [6] by using the curd-firming individual point observations recorded by a Formagraph instrument: CF_P_ (the maximum potential curd firmness at an infinite time in mm), k_CF_ (curd-firming rate constant in % × min^−^^1^), k_SR_ (syneresis rate constant in % × min^−1^), CF_max_ (maximum curd firmness in mm), and t_max_ (time to attain CFmax in min).

### 2.4. Statistical Analysis

As regards the editing of the MCP and CF_t_ traits from the 1112 milk samples, the values of RCT higher than 60 min, also called noncoagulating samples, were labeled as missing (*n* = 26); k_20_ was labeled as missing when higher than 5 min and for noncoagulating samples (*n* = 36); curd firmness was labeled as missing at 0 mm (a_30_: *n* = 29, a_45_: *n* = 24, a_60_: *n* = 26, CF_P_: *n* = 19, and CF_max_: *n* = 19).

The association analysis between the genotypes of the polymorphic SNPs with the milk composition, MCP, and CF_t_ traits was achieved using the MIXED procedure of SAS (version 9.4, SAS Inst. Inc., Cary, NC, USA), which was derived from our previous study by Dettori et al. [32]. The following model (1) was used to investigate one milk trait for each SNP at a time only for SNPs with a MAF higher than 0.05 and genotypes with a frequency higher than 0.01:

Y*_ijklm_* = µ + G*_i_* + F*_j_* + P*_k_* + DIM*_l_* + SIRE(G)*_m_* + e*_ijklm_* (1)

where Y*_ijklm_* is the observed traits of the milk composition, MCP, and CF_t_; µ is the general mean; G*_i_* is the fixed effect of the *i*th SNP genotype (*i* = 2 to 3 levels: the two homozygotes and the heterozygote); F*_j_* is the fixed effect of the *j*th farm, which also includes animal management and feeding (*j* = 1–23 levels, i.e., the different farms where animals were reared); P*_k_* is the fixed effect of the *k*th parity of the ewes (*k* = 4 levels: first, 455 sheep; second, 215 sheep; third, 212 sheep; and fourth or more parities, 230 sheep); DIM*_l_* is the fixed effect of the *l*th days in milking (*l* = 4 levels: level 1, ≤100 days, 210 sheep; level 2, 101–140 days, 381 sheep; level 3, 141–160 days, 133 sheep; and level 4, ≥161 days, 388 sheep); SIRE(G)*_m_* is the random effect of the *m*th sire (*m* = 124 different sires) nested within the genotype; and e*_ijklm_* is the error random residual effect.

The association analysis between the milk traits and the LD block was performed by a further model (2) derived from model (1), with LD*_i_* (*i* = 2 to 3 levels) instead of G*_i_* and SIRE(LD)*_m_* instead of SIRE(G)*_m_*, analyzing one milk trait for each LD block at a time.

The effects of the models were declared significant at *p* < 0.05, and multiple testing, for both models (1) and (2) and for the factors with more than two levels was performed using the Bonferroni method at α = 0.05.

## 3. Results

### 3.1. Allele Frequencies and Linkage Disequilibrium

**Figure 1:**
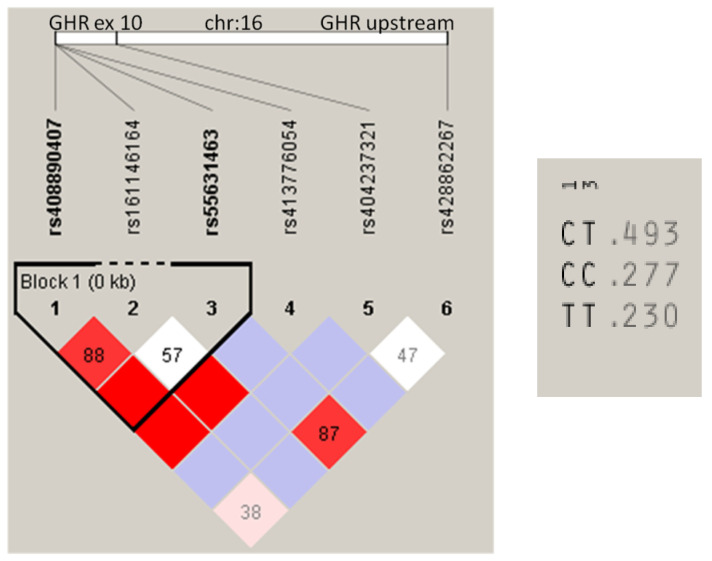
LD block defined by the SNPs genotyped in Sarda sheep (*n* = 1112), shown with haplotype frequencies on the right. LD plot of the SNPs on chromosome 16, including the GHR gene. Haploview plot of pairwise D′: red, D′ = 1.0 and logarithm of the odds (LOD) ≥ 2.0; white, D′ < 1.0 and LOD < 2.0. LD blocks are delimited by a black line. Haplotype-tagging SNPs are within black boxes. SNPs in bold are haplotype-tagging SNPs (htSNP).

The results of 45 SNPs’ genotyping are reported in Table 1. Thirty (33.3%) SNPs were polymorphic and, among these, 12 showed a MAF higher than 0.05 and were submitted to the association analysis. A linkage disequilibrium (LD) analysis was performed for all the SNPs mapping to the same chromosome. LD was highlighted in chromosome OAR:16 (Figure 1) within the *GHR* gene. The SNPs analyzed ranged from the 5′UTR region to exon 10, the last exon of the *GHR* gene. One LD block was located at exon 10 and included two htSNPs: rs408890407, synonymous mutation, and rs55631463, missense mutation. Three haplotypes were recorded with CT showing the highest frequency, 0.493, while CC and TT had 0.277 and 0.230, respectively.

### 3.2. Association Analysis

The descriptive statistics of the milk composition and coagulation traits achieved from the 1112 sampled ewes is reported in Table 2. The fixed effects included in models (1) and (2), i.e., farm, parity, and stage of lactation, showed high levels of significance, at *p* < 0.001 and *p* < 0.01, for almost all the milk traits (data not shown in the tables). The results about these effects have been widely discussed in previous papers by the same authors [5,13] using datasets connected to the present one. The proportion of variance explained by the random effect of the sire computed in both the models was always lower than 10% for almost all the SNPs and the LD block. The lowest values were recorded for the CF_p_, k_CF_, k_SR_, T_max_, and LBC, at about 0%, and the highest for the fat, protein, casein, and lactose, spanning from 5% to 9% (data not shown in the tables).

Supplementary Table S2 displays the results of the statistical analysis computed by model (1) regarding the genotype effects of the 12 polymorphic SNPs, with the MAF > 0.05, on the milk traits. The least square means of the milk yield and composition, MCP, and CF_t_, according to the genotypes with a frequency > 0.01, are reported in Table 3. SNP rs119102735 at *PRKAA2* was the only investigated polymorphic locus affecting the lactose content, and the milk samples from ewes carrying genotype TT were characterized by a lower concentration than CC and CT. The SNP with the most conspicuous effects on the milk traits was rs159573167 at *PRKAG3*, with AA homozygous ewes producing the highest daily milk yield, lower concentrations of protein and casein, the lowest value of SCS and LBC, and the shortest rennet coagulation time. SNP rs160202315 at the *TLR4* (Toll-Like Receptor 4) gene was associated with the milk composition and cell traits, milk samples from heterozygous TC ewes characterized by the best contents, i.e., higher, of protein and casein, and the highest scores of SCS and LBC. The effects of rs409504706 at *GHRHR* and rs55631463 at *GHR* were directed on the k_SR_ trait, with TT and CC ewes, respectively, showing the fastest syneresis process. SNP rs428862267 at *GHR* affected the curd firmness, and AA ewes produced milk with the highest a_30_. SNP rs160087383 at *CD14* had a highly significant effect on the milk yield (*p* < 0.001) and coagulation, and CC homozygous ewes showed both higher daily yields and better coagulation traits than TT, i.e., faster rennet coagulation times and larger curd firmness (*p* < 0.05): dMY 2012 vs. 1654 g/day, RCT 7.60 vs. 9.28 min, and a_30_ 52.52 vs. 48.03 mm, respectively.

The results of the statistical analysis computed by model (2) regarding the genotype effect of the haplotype blocks at chromosome 16 on the milk traits are reported in Supplementary Table S3. No significant effect on the investigated milk traits was found for the LD block at *GHR* (SNPs rs408890407 and rs55631463).

## 4. Discussion

### 4.1. Genes Related to Sheep Metabolism

Important metabolic variations occur during pregnancy and lactation, which involve nutrient partitioning in order to guarantee productive functions while maintaining the animal’s well-being [38]. In the present study, we evaluated the variability of genes responsible for metabolic activities and their association with milk traits in a population of Sarda sheep.

AMPK, or 5′AMP-activated protein kinase, is an enzyme (EC 2.7.11.31) formed by three protein subunits (α, β, and γ) conserved from yeast to humans. The AMPK enzyme helps to maintain the energy homeostasis of the cell, activating glucose and fatty acid uptake and their oxidation when the AMP:ATP ratio varies [39]. The alpha subunit is encoded by the *PRKAA2* gene and the gamma subunit by the *PRKAG3* gene. In the present study, we highlighted that SNP rs119102735 of the *PRKAA2* gene was associated with the milk lactose concentration. Based on the available literature, this is the first study to report a significant association between the polymorphisms at this gene and a trait regarding milk composition in dairy sheep. In accordance with our study, a recent finding by Huang et al. [40] elucidated that, in bovine mammary epithelial cells, the activation of AMP-activated protein kinase systems is linked with the reduction of lactose synthesis. The AMPK protein, composed of three subunits in humans, is expressed in different tissues, mainly in the skeletal muscle, heart, liver, and hypothalamus [41]. Hao et al. [42] did not reveal it by RNA-seq among the mRNAs expressed in the lactating mammary gland of sheep. Bionaz-Loor et al. [43] revealed, by using Real-Time PCR, that *PRKAA2* is expressed in the mammary glands of lactating cattle, and the same authors did not reveal variations in the expression of this gene during the analyzed period. They observed that, although high-protein synthesis persisted during lactation, the level of mRNA synthesis for the AMPK subunits, or other genes corresponding to related biosynthetic pathways, did not vary. In sheep, Crisà et al. [44] analyzed a polymorphism in exon 4 of *PRKAA2*, which was not associated with changes in the synthesis of fatty acids in milk. AMPK is a molecule that functions as a metabolic switch, which, in the absence of ATP, blocks consumption and activates the catabolic pathways to produce ATP, and turns off the anabolic pathways, which consume ATP [45]. Since AMPK is involved in linking energy sensing to the metabolic pathways, it plays an important role at the whole-body level by translating endocrine communications into adapted metabolic responses [46]. For this reason, it is possible that the observed association between *PRKAA2* and lactose might not to be directly linked to a variation of the AMPK expression or activity in the secretory cell of the mammary gland but, rather, to the regulation of the animal’s metabolism in the physiological phase of lactation.

We also genotyped three SNPs of the *PRKAG3* gene and evidenced that SNP rs159573167 AA was associated with an increase of dMY in the sampled population (+17% in comparison with genotype GG; data not shown in the tables) and a decrease of the protein and casein contents. The variability at the *PRKAG3* gene was investigated in New Zealand Suffolk sheep [47], and the variability and expression profile in skeletal muscle were investigated in five local Spanish breeds, where *PRKAG3* was found to be highly expressed [48]. Here, for the first time, we highlighted an association between *PRKGA3* and dMY and the protein and casein contents. Milk from *PRKAG3* rs159573167 AA ewes also showed the lowest SCS and LBC scores, which was not directly explained by its function, and the rennet coagulation time (RCT). In the present study, the occurrence of lower concentrations of protein, somatic, and bacterial cells and higher daily yields found in *PRKAG3* rs159573167AA ewes might be attributable, in addition to the genetic effect, to the so-called dilution effect, as the higher the milk yield, the lower the level of total solids and cells and vice versa [1].

We also genotyped twelve SNPs from genes *IGF1*, *GHRHR*, and *GHR*, which are part of the growth hormone pathway and play an important role in lipid, protein, and glucose metabolism [27]. We highlighted an association with curd firmness a_30_ (*GHR* rs428862267) and the syneresis instant rate constant, k_SR_ (*GHR* rs55631463 and *GHRHR* rs409504706). Previous studies, carried out in a subset of the resource sheep population, have revealed an association of several SNPs at the *GHR* gene with the daily fat and protein yields [49] and with the k_SR_ [50]. The k_SR_ is a derived parameter depending on the speed of syneresis, which, in turn, is related to the lipid content of the milk [7].

### 4.2. Genes Related to Innate Immunity

The role of specific genes in the innate immune response to some pathogens in ruminants has been investigated [51], due to its potential positive impact in sustainable agriculture systems. Here, we evaluated the association of some genes involved in innate immunity with milk traits in dairy Sarda sheep. Genotype CC at SNP rs160087383 of the *CD14* gene was associated with an increase of dMY. Li et al. [52] investigated the role of the *CD14* gene in relation to the Gram-negative bacteria-induced innate immune response and revealed that the variability of this gene is associated with the morbidity of Chinese Holsteins. Thus, the *CD14* gene, which is expressed in macrophages and plays an important role in a number of pathways related to the immune response, in our set of animals was found to be associated with the daily milk yield. The *CD14* rs160087383 CC ewes were characterized by the highest daily milk yield and the concurrence of favorable coagulation traits, i.e., a faster RCT and a larger curd firmness a_30_. As observed also for the *PRKAG3* gene, the milk samples from high-yielding ewes were characterized by the shortest, and consequently better, values of the rennet coagulation time. This positive feature is in accordance with a study using a dataset interconnected with the present one [5] that has evidenced this peculiar result regarding sheep species, because cow milk yields and RCT commonly show a positive (i.e., unfavorable) correlation [53].

In our investigation, SNP rs160202315 at the *TLR4* gene was associated with both the protein and cellular traits. The significant effect of the *TLR4* gene on the protein and casein contents is analogous to that recorded for cattle species [54], while effects on milk coagulation have been found for *TLR2*, encoding for a protein of the same family, which influences the curd-firming time in dairy cows [55]. Milk samples from *TLR4* rs160202315 CC homozygous ewes were characterized by the lowest scores of SCS and LBC; this association can be explained in relation to the gene function, pathogen recognition, and innate immunity, as described above.

We have to keep in mind that the observed associations between the genotyped SNPs and dairy traits may be due to linkages with unknown causal variations.

## 5. Conclusions

The designed open array revealed some new genetic patterns and the related influences on the milk yield, composition, and coagulation traits. The results obtained in the present study from the association analysis evidenced several significant effects of the investigated SNPs on the milk traits, and these may represent an advancement for the dairy sheep sector. In particular, for the Sarda sheep breed, this information might be useful in appropriate breeding schemes, but these should be confirmed by further studies, possibly performed on independent populations.

## Figures and Tables

**Table 1:** The variability of 45 SNPs genotyped in Sarda sheep (*n* = 1112).

Gene SNP ID	ObsH	PredH	HWpv	%Gen	MAF	Alleles
*PRKAA2:* Protein Kinase AMP-Activated Catalytic Subunit Alpha2						
rs119102735	0.319	0.316	0.91	96.6	0.197	C:(T)
rs159701443	0.004	0.004	1.00	97.8	0.002	G:(A)
rs159701441	0.161	0.157	0.57	86.2	0.086	T:(C)
rs159701440	0.011	0.019	0.01	91.8	0.009	G:(A)
*PRKAG3:* Protein Kinase AMP-Activated Non-Catalytic Subunit Gamma3						
rs159573140	0.000	0.000	1.00	100	0.000	G:G
rs159573109	0.001	0.001	1.00	99.0	0.000	T:T
rs159573167	0.008	0.435	0.01	96.8	0.320	G:(A)
*B4GALT1:* Beta-1,4-Galactosyltransferase 1						
rs160176029	0.013	0.018	0.96	98.6	0.009	G:(A)
rs160176020	0.000	0.000	1.00	99.9	0.000	A:A
rs160175821	0.000	0.000	1.00	100	0.000	A:A
rs160175809	0.419	0.415	0.85	79.5	0.294	C:(T)
*TLR4:* Toll Like Receptor 4						
rs160202330	0.000	0.000	1.00	99.3	0.000	A:A
rs160202321	0.005	0.005	1.00	99.5	0.003	A:(C)
rs160202315	0.053	0.407	0.01	97.5	0.284	T:(C)
*TYRP1:* Tyrosinase Related Protein 1						
rs416417209	0.323	0.335	0.31	96.6	0.212	T:(C)
*IGF1:* Insulin Like Growth Factor 1						
rs159876390	0.339	0.459	0.01	86.7	0.356	G:(A)
rs159876394	0.002	0.002	1.00	99.5	0.001	C:(G)
rs401028781	0.000	0.000	1.00	98.8	0.000	G:G
*GHRHR:* Growth Hormone Releasing Hormone Receptor						
rs409504706	0.314	0.343	0.01	95.5	0.220	T:(G)
rs161797246	0.004	0.004	1.00	94.5	0.002	C:(T)
*CD14:* Cluster of Differentiation 14 Molecule						
rs160087365	0.001	0.001	1.00	98.3	0.000	C:C
rs160087371	0.001	0.008	0.01	99.2	0.004	G:(C)
rs160087378	0.000	0.000	1.00	99.0	0.000	C:C
rs160087383	0.003	0.121	0.01	99.4	0.064	T:(C)
*ABCG2:* ATP Binding Cassette Subfamily G Member 2						
rs159956845	0.000	0.0	1.00	99.3	0.000	T:T
rs159956885	0.004	0.005	0.01	98.9	0.003	A:(G)
rs159956974	0.002	0.023	0.01	87.4	0.011	T:(G)
*NPFFR2:* Neuropeptide FF Receptor 2						
rs159980590	0.034	0.033	1.00	91.5	0.017	A:(C)
rs159980593	0.013	0.015	0.11	96.9	0.007	T:(A)
*CSNK1G1:* Casein Kinase 1 Gamma 1						
rs160322386	0.000	0.000	1.00	100	0.000	G:G
*GHR:* Growth Hormone Receptor						
rs408890407	0.326	0.354	0.01	96.3	0.230	C:(T)
rs161146164	0.012	0.012	1.00	98.7	0.006	T:(G)
rs55631463	0.395	0.399	0.79	96.7	0.276	T:(C)
rs413776054	0.010	0.010	1.00	99.5	0.005	G:(A)
rs404237321	0.012	0.012	1.00	99.5	0.006	C:(T)
rs428862267	0.448	0.490	0.01	94.4	0.430	G:(A)
rs425834583	0.000	0.000	1.00	100	0.000	A:A
*TRL2:* Toll Like Receptor 2						
rs162073318	0.001	0.001	1.00	99.5	0.000	C:C
*PI:* Serpin Peptidase Inhibitor						
rs397514170	0.000	0.000	1.00	100	0.000	A:A
rs397514169	0.001	0.001	1.00	99.5	0.000	G:G
rs397514168	0.000	0.000	1.00	99.7	0.000	T:T
rs397514150	0.002	0.002	1.00	98.9	0.001	T:(C)
*PLCE1:* Phospholipase C Epsilon						
rs161473124	0.061	0.059	0.73	99.0	0.030	C:(T)
rs161473126	0.001	0.003	0.01	99.0	0.001	T:(C)
rs161473140	0.006	0.006	1.00	90.9	0.003	G:(A)

ObsH: observed heterozygosity, PredH: predicted heterozygosity, HWpv: Hardy-Weinberg test *p*-value, %Gen: percentage of genotyped samples, MAF: minor allele frequency, and minor alleles in brackets.

**Table 2:** Descriptive statistics of the milk yield and composition, milk coagulation properties (MCP), and curd firmness over time traits (CF_t_) from the sampled population of Sarda sheep (*n* = 1112).

Milk Traits	Mean	SD	Min	Max
Milk yield and composition				
dMY (g/day)	1597	842	181	5760
Fat (g/100 mL)	6.44	1.19	1.93	12.52
Protein (g/100 mL)	5.41	0.65	3.52	8.24
Casein (g/100 mL)	4.21	0.54	2.45	6.67
Lactose (g/100 mL)	4.81	0.31	2.40	5.59
pH	6.67	0.09	6.45	7.14
SCC (*n* × 1000/mL)	1253	2993	5	20,612
TBC (*n* × 1000/mL)	2522	4872	1	17,000
MCP				
RCT (min)	8.94	4.92	1.30	52.30
k_20_ (min)	1.98	0.79	1.30	11.30
a_30_ (mm)	49.48	12.99	0	70.00
a_45_ (mm)	45.46	15.17	0	73.88
a_60_ (mm)	41.84	16.56	0	77.98
CF_t_				
CF_P_ (mm)	62.1	316.2	0	360.8
k_CF_ (% × min^−1^)	27.0	13.0	1.0	90.0
k_SR_ (% × min^−1^)	1.5	1.9	0.1	16.3
CF_max_ (mm)	54.17	9.96	0	75.96
t_max_ (min)	30.57	13.41	8.75	60

dMY: daily milk yield; SCC: somatic cell count; TBC: total bacterial count; RCT: rennet coagulation time; k_20_: curd-firming time; a_30_, a_45_, and a_60_: curd firmness 30, 45, and 60 min after rennet addition; CF_P_: the maximum potential curd firmness after an infinite time; k_CF_: curd-firming rate constant; k_SR_: syneresis rate constant; CF_max_: maximum curd firmness; and t_max_: time to attain CF_max_.

**Table 3:** Least square means of the milk yield and composition, the milk coagulation properties (MCP), and curd firmness over time traits (CF_t_), according to the genotypes of the SNPs investigated in the Sarda sheep (*n* = 1112).

		Milk Yield and Composition						MCP		CF_t_
Gene SNP ID ^#^	Genotype and (*n*)	dMY	Protein	Casein	Lactose	SCS	LBC	RCT	a_30_	k_SR_
*PRKAA2* rs119102735	CC (695)	1681	5.39	4.20	**4.79 ^b^**	4.84	2.42	9.12	48.47	1.46
	CT (340)	1657	5.36	4.17	**4.79 ^b^**	4.93	2.38	9.08	47.94	1.65
	TT (41)	1679	5.42	4.23	**4.68 ^a^**	5.03	2.28	9.26	45.47	1.46
*PRKAG3* rs159573167	AA (346)	**1859 ^B^**	**5.24 ^a^**	**4.09 ^a^**	4.84	**4.32 ^A^**	**1.77 ^A^**	**8.03 ^a^**	49.20	1.18
	AG (11)	**1851 ^AB^**	**5.49 ^b^**	**4.29 ^b^**	4.84	**4.85 ^B^**	**2.22 ^AB^**	**10.79 ^b^**	47.73	1.82
	GG (724)	**1585 ^A^**	**5.45 ^b^**	**4.25 ^b^**	4.76	**5.17 ^B^**	**2.62 ^B^**	**9.77 ^b^**	47.71	1.64
*TLR4* rs160202315	CC (286)	1747	**5.21 ^a^**	**4.06 ^a^**	4.85	**4.26 ^a^**	**1.63 ^A^**	8.35	47.87	1.02
	TC (58)	1742	**5.53 ^b^**	**4.32 ^b^**	4.76	**4.93 ^b^**	**2.50 ^B^**	9.77	48.38	1.95
	TT (746)	1637	**5.44 ^b^**	**4.24 ^ab^**	4.76	**5.15 ^b^**	**2.61 ^B^**	9.50	48.52	1.71
*GHRHR* rs409504706	GG (67)	1645	5.33	4.15	4.76	5.32	2.31	9.73	47.62	**2.14 ^b^**
	GT (334)	1684	5.36	4.18	4.79	4.79	2.47	8.92	47.86	**1.58 ^ab^**
	TT (662)	1680	5.38	4.19	4.79	4.85	2.36	9.14	48.43	**1.42 ^a^**
*CD14* rs160087383 ^§^	CC (70)	**2012 ^B^**	5.32	4.15	4.81	4.40	2.03	**7.60 ^a^**	**52.52 ^b^**	1.45
	TT (1037)	**1654 ^A^**	5.38	4.20	4.78	4.90	2.39	**9.28 ^b^**	**48.03 ^a^**	1.50
*GHR* rs55631463	CC (85)	1743	5.36	4.18	4.78	5.00	2.33	9.36	48.61	**1.11 ^A^**
	CT (426)	1645	5.37	4.19	4.77	4.82	2.41	9.38	47.38	**1.79 ^B^**
	TT (567)	1680	5.38	4.19	4.79	4.94	2.39	8.98	48.70	**1.39 ^A^**
*GHR* rs428862267	AA (214)	1737	5.39	4.19	4.77	4.93	2.27	8.88	**50.21 ^b^**	1.28
	GA (470)	1648	5.37	4.19	4.80	4.80	2.32	8.88	**47.95 ^a^**	1.61
	GG (366)	1675	5.36	4.17	4.79	4.90	2.47	9.50	**48.05 ^ab^**	1.51

^#^ SNPs out of the 45 investigated with MAP > 0.05, genotypes with frequencies > 0.01, and at least one significant *p*-value in one of the 18 investigated milk traits. ^§^ Genotype CT not computed because of the frequency < 0.01. dMY: daily milk yield in g/day. Protein, casein, and lactose in g/100 mL. SCS: somatic cell score = log_2_ (SCC × 10^−5^) + 3. LBC: logarithmic bacterial count = log_10_ total bacterial count (total bacterial count/1000). RCT: rennet coagulation time in min. a_30_: curd firmness 30 min after rennet addition in mm. k_SR_: syneresis rate constant in % × min^−1^. ^a,b^ Different lower-case letters for the same SNP differ significantly at *p* < 0.05. ^A,B^ Different upper-case letters for the same SNP differ significantly at *p* < 0.01. Least square means for the milk traits with statistical significance are in bold.

## Data Availability

Not applicable.

## Supplementary Materials

The following are available online at https://www.mdpi.com/article/10.3390/ani11082207/s1: Table S1: Gene names, SNP ID, and context sequences of the 45 SNPs genotyped in the sampled population of Sarda sheep (*n* = 1112). Table S2: *p*-value and significance for the milk yield and composition, milk coagulation properties (MCP), and curd firmness over time traits (CF_t_), according to the effects of 12 polymorphic SNPs out of 45 investigated in Sarda sheep (*n* = 1112). Table S3: *p*-value and significance for the milk yield and composition, milk coagulation properties (MCP), and curd firmness over time traits (CF_t_), according to the effects of the block evidenced among the 45 SNPs investigated in Sarda sheep (*n* = 1112).
