# Supplementary material for: Novel Genes Associated with Dairy Traits in Sarda Sheep
Source: Animals (Basel). 2021 Jul 26;11(8):2207. doi: 10.3390/ani11082207 (PMC8388407; doi:10.3390/ani11082207)
Supplement: Supplementary file 1 [file animals-11-02207-s001.zip › animals-1286244-SI.pdf]

**Table S1.** Gene names, SNP ID and context sequences of the 45 SNPs genotyped in the sampled population of Sarda sheep (n = 1,112)

| Gene symbol | SNP ID | Chr position | Context sequence |
|---|---|---|---|
| Protein Kinase AMP-Activated Catalytic Subunit Alpha2 - *PRKAA2* | rs119102735 | 1:30924815 | GGCTCTTTCAGCAGATTCTGTCTGC[C/T]GTGGATTACTGTCATAGGCATATGG |
| | rs159701443 | 1:30927608 | TCCAGATATCCACTTCAGGACCTGC[A/G]TACAGCCTAGGGGAAGATGACAGCA |
| | rs159701441 | 1:30927701 | CACCCCCTCGGATCTTCTTAAACAG[C/T]GTAGGCACATGCTCATCATCAAATG |
| | rs159701440 | 1:30927764 | CCTGCAGCATATGCATCAGGAGAGT[A/G]GCAACAGAACGATTGAGATATTCTG |
| Protein Kinase AMP-Activated Non-Catalytic Subunit Gamma3 - *PRKAG3* | rs159573140 | 2:219779811 | GATGCCCAGATCTTGGATGGTGCGG[G/T]AGAGGAAGGAGGGCCGGGGCAGCAG |
| | rs159573109 | 2:219781592 | TCCCTCCAGACACAGCGTCCTCCGC[C/T]TCAGTGCTTCTCCCACACTTATATC |
| | rs159573167 | 2:219782111 | AGCCTGAGCACCGGCACCTGCGGGG[A/G]CGGGCACTGGGCCGGCCTCTCTTCC |
| Beta-1,4-Galactosyltransferase 1 - *B4GALT1* | rs160176029 | 2:37813170 | GTTCTGCAAAGGCGGCGGCGGTGCG[A/G]CCCCTCGCAGCCGGAGCTCCCCGGA |
| | rs160176020 | 2:37813323 | GAGCAGCGGGGACTCCTCAGGGCAT[A/C]CGGTCAGCGAGCGTGTTGTGGTGGA |
| | rs160175821 | 2:37862428 | ATTAGGTTCATTTTTCTTGTCTCTC[A/G]AGTGGCGGATCATCCGACACTTCCC |
| | rs160175809 | 2:37863227 | CAAGTTGGGGGCAATATATCCCACT[C/T]GTCCTGGTCGTCCGGAAAGGCGTCT |
| Toll Like Receptor 4 - *TLR4* | rs160202330 | 2:5881259 | CAGGTGGAGCTCTATCGCCTTCTGA[A/G]CAGGAACACCTACCTGGAGTGGGAG |
| | rs160202321 | 2:5881892 | ACTTGGCTAAATCTTACTCAGAATG[A/C]ATTTGCTTGTGTTTGTGAACATCAG |
| | rs160202315 | 2:5882073 | AGCCCAGACAGCATTTCACTCCCTC[C/T]CTAGCCTTCAGGTGCTGAATATGAG |
| Tyrosinase Related Protein 1 - *TYRP1* | rs416417209 | 2:80645322 | CCTGGGCTCAGTTCCCGAGAGAGTG[C/T]GCTACCATTGAGGCTTTGAGGAATG |
| Insulin Like Growth Factor 1 - *IGF1* | rs159876390 | 3:171055347 | GGGGCGCCCTCCGACTGCTCGAGCC[A/G]TACCCCGTGGGCTTGTCTGTTCAGA |
| | rs159876394 | 3:171108350 | CTCGGGTCCCGCCGTGGCAGAGCTG[C/G]TGAAGGCGAGCAAGCACAGGGCCAG |
| | rs401028781 | 3:171113596 | GCAACCAGGACGAGGGGTCATCCCA[G/C]CGCCGTCTTCCAGTCTAGTTTACCC |
| Growth Hormone Releasing Hormone Receptor - *GHRHR* | rs409504706 | 4:65329776 | AATCTGGGAGGACTTTCTGGAAGAG[G/T]TGTCCTTGAATTAGACTTTGAAAGA |
| | rs161797246 | 4:65363733 | CATGGGGCAGCCCTGAGACCCCTTA[C/T]CCAGTGGTCCTGGAAATTGTGCGTA |
| Cluster of Differentiation 14 Molecule - *CD14* | rs160087365 | 5:49076357 | ACCACGCCGGAGATCATCGGGTCAT[C/T]TTGGTGCTGGAGGGCTCCAGGGTCC |
| | rs160087371 | 5:49076541 | GCCCAGCGAACGACAAATTGAGAGA[C/G]CTTAGTGCACTGGGCCAGACACATC |
| | rs160087378 | 5:49076911 | TCGCCGAGCCAGGCACCTCCTGTTG[C/T]CCACGATACGTTACGGAGACTGAGG |
| | rs160087383 | 5:49077142 | ATTGTGTCAGCATACTGCTTCGGGT[C/T]GTGTCGGCTCCCTTGAGAAACTGTT |
| ATP Binding Cassette Subfamily G Member 2 -*ABCG2* | rs159956845 | 6:36443358 | TTCCCTCCACAGGAAGGCGGAAATG[C/T]TCAAAATGTCTTCCAATAGTTACGA |
| | rs159956885 | 6:36467030 | TGTCATCAGCTCAAATGGATTTCCA[A/G]GCGTTCATTCAAAAATTTACTGGGT |
| | rs159956974 | 6:36472926 | TTTCTATGATCTAAAAAATGATCCT[G/T]CAGGAATCCAGAACAGGTGAGTAAA |

| | | | |
|---|---|---|---|
| Neuropeptide FF Receptor 2 - *NPFFR2* | rs159980590 | 6:86895625 | AACTGGCATTACATTTGGAATAATG[A/C]CACAACACATGATCTGTACTCAGAT |
| | rs159980593 | 6:86895777 | CACAGTGGTTTGCTTCATTGTAATG[A/T]GGAACAAACATATGCACACAGTCAC |
| Casein Kinase 1 Gamma 1 - *CSNK1G1* | rs160322386 | 7:42831179 | AATACATTGACCCTGAAACCAAAAA[A/G]CATATACCTTATAGGGAACACAAAA |
| Growth Hormone Receptor – *GHR* | rs408890407 | 16:31833017 | CTTTGTCAGGCAAGGGCAGGGCAGT[C/T]GCATTGAGTACGAGGCCCTGTGGAG |
| | rs161146164 | 16:31833100 | ACAGGTATCTCAGAACTTGGAACAT[T/G]TTCTGCTGTCCCTGACCTCCCGGCT |
| | rs55631463 | 16:31833271 | AAGTAAGCGCTGTCCACGATGAAGT[C/T]TGCTTGGCTGGGTGTGACCACTTCT |
| | rs413776054 | 16:31833547 | CTAGCAGGGGCAGCATCATTAGAAG[G/A]TGAGTTATTTTGATTCTTCTGATCA |
| | rs404237321 | 16:31859509 | AGAGAAACACTTATGATCCACAATA[C/T]CACCATTGCTAGTTAGCTTGATGCA |
| | rs428862267 | 16:32001143 | CACTGATACTTTTCCAGGGCTATAG[G/A]AATTACTATCTAACTATCATAGTTG |
| | rs425834583 | 16:32003365 | GGACAGGCACTATAGTATTTCCAGT[A/C]CCGAGGGCCTGTCAAGGACGGTGAT |
| Toll Like Receptor 2 - *TLR2* | rs162073318 | 17:3842561 | ATCCACGGGCCACTCCAGGTAGGTC[C/T]TTGTCAATGGGCTCCAGCAGAATCA |
| Serpin Peptidase Inhibitor – *PI* | rs397514170 | 18:57828567 | CTGCAGCAGCTGGAAGACAAGCTCA[A/G]CAACGAACTCCTCGCCAAGTTCCTG |
| | rs397514169 | 18:57828697 | GAAGGTGCCCATGATGAACCGCCTG[A/G]GCATGTTTGACCTCCACTACTGTGA |
| | rs397514168 | 18:57828741 | CACACCACGGAGAGGGACTTCCACG[C/T]GAATGAGCAAACCACCGTGAAGGTG |
| | rs397514150 | 18:57829882 | CTGCAACTGACCACCGGCAATGGTC[C/T]GTTCATCAATGAGAGTGCAAAGCTA |
| Phospholipase C Epsilon - 1*PLCE1* | rs161473124 | 22:14967835 | CAGTGACGAGAACAGTAATGAAAAG[C/T]GCTGGGAGAAAAGCATGCCAGATTC |
| | rs161473126 | 22:14967919 | ATTGAAAAACCATCAGCACGGCCCT[C/T]CTCAGAGCCAGTTTTATGAAACGTG |
| | rs161473140 | 22:14968714 | GAGAGTCATGGAACGGCTGTCAACA[A/G]TGGTGGTTAGGCAAGATGGTTCCCA |

SNP ID: dbSNP reference records; Chr pos.: chromosome position on Oar_v3.1.

**Table S2.** *p*-value and significance for milk yield and composition, milk coagulation properties (MCP) and curd firmness over time traits ($CF_t$) according to the effect the 12 polymorphic SNPs out of 45 investigated in Sarda sheep (n = 1,112).

| Gene | SNP ID | Milk yield and composition | | | | | | | | MCP | | | | | $CF_t$ | | | | |
|---|---|---|---|---|---|---|---|---|---|---|---|---|---|---|---|---|---|---|---|
| | | dMY | Fat | Protein | Casein | Lactose | pH | SCS | LBC | RCT | $k_{20}$ | $a_{30}$ | $a_{45}$ | $a_{60}$ | $CF_P$ | $k_{CF}$ | $k_{SR}$ | $CF_{max}$ | $t_{max}$ |
| *PRKAA2* | rs119102735 | 0.773 | 0.202 | 0.706 | 0.611 | 0.045* | 0.714 | 0.735 | 0.808 | 0.971 | 0.866 | 0.255 | 0.194 | 0.588 | 0.477 | 0.842 | 0.406 | 0.180 | 0.928 |
| *PRKAA2* | rs159701441 | 0.934 | 0.455 | 0.729 | 0.713 | 0.420 | 0.614 | 0.775 | 0.450 | 0.747 | 0.684 | 0.960 | 0.684 | 0.517 | 0.923 | 0.406 | 0.470 | 0.319 | 0.557 |
| *PRKAG3* | rs159573167 | 0.001*** | 0.194 | 0.015* | 0.023* | 0.065 | 0.898 | 0.004** | 0.001*** | 0.019* | 0.676 | 0.608 | 0.998 | 0.665 | 0.563 | 0.159 | 0.248 | 0.763 | 0.857 |
| *B4GALT1* | rs160175809 | 0.936 | 0.334 | 0.339 | 0.470 | 0.753 | 0.673 | 0.417 | 0.631 | 0.769 | 0.528 | 0.798 | 0.723 | 0.332 | 0.424 | 0.765 | 0.418 | 0.408 | 0.999 |
| *TLR4* | rs160202315 | 0.102 | 0.216 | 0.016* | 0.022* | 0.240 | 0.387 | 0.023* | 0.001*** | 0.328 | 0.865 | 0.940 | 0.672 | 0.642 | 0.837 | 0.061 | 0.122 | 0.131 | 0.962 |
| *TYRP1* | rs416417209 | 0.617 | 0.432 | 0.383 | 0.557 | 0.980 | 0.666 | 0.800 | 0.161 | 0.346 | 0.316 | 0.674 | 0.807 | 0.473 | 0.760 | 0.424 | 0.671 | 0.825 | 0.492 |
| *IGF1* | rs159876390 | 0.457 | 0.379 | 0.223 | 0.182 | 0.800 | 0.551 | 0.090 | 0.060 | 0.834 | 0.830 | 0.401 | 0.275 | 0.326 | 0.106 | 0.436 | 0.504 | 0.916 | 0.824 |
| *GHRHR* | rs409504706 | 0.814 | 0.576 | 0.692 | 0.655 | 0.817 | 0.286 | 0.128 | 0.442 | 0.415 | 0.205 | 0.713 | 0.246 | 0.202 | 0.837 | 0.088 | 0.030* | 0.693 | 0.618 |
| *CD14* | rs160087383 | 0.001*** | 0.848 | 0.523 | 0.530 | 0.603 | 0.862 | 0.235 | 0.215 | 0.039* | 0.279 | 0.019* | 0.112 | 0.275 | 0.531 | 0.775 | 0.879 | 0.130 | 0.895 |
| *GHR* | rs408890407 | 0.544 | 0.835 | 0.849 | 0.902 | 0.065 | 0.389 | 0.372 | 0.729 | 0.498 | 0.241 | 0.773 | 0.999 | 0.962 | 0.161 | 0.289 | 0.337 | 0.944 | 0.436 |
| *GHR* | rs55631463 | 0.140 | 0.653 | 0.930 | 0.988 | 0.780 | 0.296 | 0.596 | 0.853 | 0.443 | 0.308 | 0.214 | 0.130 | 0.349 | 0.387 | 0.109 | 0.001*** | 0.767 | 0.764 |
| *GHR* | rs428862267 | 0.094 | 0.600 | 0.822 | 0.808 | 0.373 | 0.666 | 0.658 | 0.214 | 0.131 | 0.763 | 0.039* | 0.065 | 0.207 | 0.705 | 0.330 | 0.159 | 0.395 | 0.454 |

dMY: daily milk yield; SCS: somatic cell score = $\log_2(SCC \times 10^{-5}) + 3$; LBC: logarithmic bacterial count = $\log_{10}$ total bacterial count (total bacterial count/1,000); RCT: rennet coagulation time; $k_{20}$: curd firming time; $a_{30}$, $a_{45}$, and $a_{60}$: curd firmness 30, 45 and 60 minutes after rennet addition; $CF_P$: the maximum potential curd firmness after an infinite time; $k_{CF}$: curd-firming rate constant; $k_{SR}$: syneresis rate constant; $CF_{max}$: maximum curd firmness; and $t_{max}$: time to attain $CF_{max}$.

*** $p < 0.001$; ** $p < 0.01$; * $p < 0.05$; no asterisk: non significant.

**Table S3.** *p*-value and significance for milk yield and composition, milk coagulation properties (MCP) and curd firmness over time traits ($CF_t$) according to the effect of the block evidenced among the 45 SNPs investigated in Sarda sheep (n = 1,112).

| Block ID | Milk yield and composition | | | | | | | | MCP | | | | | $CF_t$ | | | | |
|---|---|---|---|---|---|---|---|---|---|---|---|---|---|---|---|---|---|---|
| | dMY | Fat | Protein | Casein | Lactose | pH | SCS | LBC | RCT | $k_{20}$ | $a_{30}$ | $a_{45}$ | $a_{60}$ | $CF_P$ | $k_{CF}$ | $k_{SR}$ | $CF_{max}$ | $t_{max}$ |
| Block 1 | 0.870 | 0.429 | 0.989 | 0.803 | 0.320 | 0.689 | 0.954 | 0.326 | 0.695 | 0.894 | 0.579 | 0.536 | 0.838 | 0.325 | 0.507 | 0.572 | 0.967 | 0.889 |

Block 1: chromosome 16, GHR gene SNPs rs408890407 and rs55631463. dMY: daily milk yield; SCS: somatic cell score = $\log_2$ (SCC × $10^{-5}$) + 3; LBC: logarithmic bacterial count = $\log_{10}$ total bacterial count (total bacterial count/1,000); RCT: rennet coagulation time; $k_{20}$: curd firming time; $a_{30}$, $a_{45}$, and $a_{60}$: curd firmness 30, 45 and 60 minutes after rennet addition; $CF_P$: the maximum potential curd firmness after an infinite time; $k_{CF}$: curd-firming rate constant; $k_{SR}$: syneresis rate constant; $CF_{max}$: maximum curd firmness; and $t_{max}$: time to attain $CF_{max}$.

No asterisk: non significant.
